# RETRACTION: HSP27 Associates With Epithelial–Mesenchymal Transition, Stemness and Radioresistance of Salivary Adenoid Cystic Carcinoma

**DOI:** 10.1111/jcmm.70365

**Published:** 2025-01-13

## Abstract

**RETRACTION:** W. Chen, X. Ren, J. Wu, X. Gao, X. Cen, S. Wang, S. Sheng, Q. Chen, Y.‐J. Tang, X.‐H. Liang, and Y.‐L. Tang, “HSP27 Associates with Epithelial–Mesenchymal Transition, Stemness and Radioresistance of Salivary Adenoid Cystic Carcinoma,” *Journal of Cellular and Molecular Medicine* 22, no. 4 (2018): 2283–2298, https://doi.org/10.1111/jcmm.13510.

The above article, published online on 09 February 2018 in Wiley Online Library (wileyonlinelibrary.com), has been retracted by agreement between the journal Editor‐in‐Chief, Stefan Constantinescu; The Foundation for Cellular and Molecular Medicine; and John Wiley and Sons Ltd. The retraction has been agreed upon following an investigation into concerns raised by a third party, which revealed inappropriate image panel duplications within the article depicting different experimental conditions (Figure 1B Control and EV, Figure 2C Control and Neg, Figure 3A Control and EV, Figure 1F SACC‐LM Control and CM+HSP27, and Figure 6B SACC‐LM CM without IR and CM with IR panels). Additionally, irregularities were discovered regarding the appearance of the dissected tumors in Figure 5A and another instance of image duplication was identified between Figure 1F SACC‐LM Control panel and another image published previously by an overlapping group of authors in a different scientific context. The explanation provided by the authors was unsatisfactory and the partial raw data shared could not address these concerns. Thus, the editors have lost confidence in the presented data and consider the conclusions of this manuscript substantially compromised. The authors disagree with the retraction wording.

**RETRACTION:** W. Chen, X. Ren, J. Wu, X. Gao, X. Cen, S. Wang, S. Sheng, Q. Chen, Y.‐J. Tang, X.‐H. Liang, and Y.‐L. Tang, “HSP27 Associates with Epithelial–Mesenchymal Transition, Stemness and Radioresistance of Salivary Adenoid Cystic Carcinoma,” *Journal of Cellular and Molecular Medicine* 22, no. 4 (2018): 2283–2298, https://doi.org/10.1111/jcmm.13510.

The above article, published online on 09 February 2018 in Wiley Online Library (wileyonlinelibrary.com), has been retracted by agreement between the journal Editor‐in‐Chief, Stefan Constantinescu; The Foundation for Cellular and Molecular Medicine; and John Wiley and Sons Ltd. The retraction has been agreed upon following an investigation into concerns raised by a third party, which revealed inappropriate image panel duplications within the article depicting different experimental conditions (Figure 1B Control and EV, Figure 2C Control and Neg, Figure 3A Control and EV, Figure 1F SACC‐LM Control and CM+HSP27, and Figure 6B SACC‐LM CM without IR and CM with IR panels). Additionally, irregularities were discovered regarding the appearance of the dissected tumors in Figure 5A and another instance of image duplication was identified between Figure 1F SACC‐LM Control panel and another image published previously by an overlapping group of authors in a different scientific context. The explanation provided by the authors was unsatisfactory and the partial raw data shared could not address these concerns. Thus, the editors have lost confidence in the presented data and consider the conclusions of this manuscript substantially compromised. The authors disagree with the retraction wording.

